# *In Vitro* Evaluation of the Antimicrobial Efficacy of Four Endodontic Biomaterials against *Enterococcus faecalis*, *Candida albicans*, and *Staphylococcus aureus*

**DOI:** 10.1155/2014/383756

**Published:** 2014-10-12

**Authors:** Duddi Narendra Nirupama, Mohan Thomas Nainan, Rajendran Ramaswamy, Sethumadhavan Muralidharan, Hulimangala Hosakote Lingareddy Usha, Roshni Sharma, Soham Gupta

**Affiliations:** ^1^Department of Conservative Dentistry & Endodontics, Vydehi Institute of Dental Sciences & Research Centre, No. 82, EPIP Area, Nallurahalli, Whitefield, Bangalore, Karnataka 560066, India; ^2^Department of Microbiology, Vydehi Institute of Medical Sciences & Research Centre, No. 82, EPIP Area, Nallurahalli, Whitefield, Bangalore, Karnataka 560066, India; ^3^Department of Microbiology, St. John's Medical College, Sarjapur Road, Bangalore, Karnataka 560034, India; ^4^Department of Conservative Dentistry & Endodontics, V. S. Dental College & Research Centre, Bangalore, Karnataka 560001, India; ^5^Department of Conservative Dentistry & Endodontics, Army College of Dental Sciences, Secunderabad 500087, India

## Abstract

Root canal sealers that possess good antimicrobial property can prevent residual and recurrent infection and contribute to successful endodontic therapy. This study evaluated the antimicrobial activity of four endodontic sealers, AH Plus, Tubliseal EWT, EndoRez, and iRoot SP, against three different microorganisms, *E. faecalis, C. albicans*, and *S. aureus*, by direct contact test. 10 *μ*L microbial suspensions were allowed to directly contact the four endodontic sealers for 1 hr at 37°C. Subsequently microbial growth was measured spectrophotometrically every 30 min for 18 hours. The microbial suspensions were simultaneously tested to determine the antimicrobial effect of components which are capable of diffusing into the medium. The results revealed that AH Plus and iRootSP had significantly higher antimicrobial activity against *E. faecalis*. AH Plus and Tubliseal EWT showed significantly higher antimicrobial activity against *C. albicans* and *S. aureus* compared to iRoot SP and EndoRez. EndoRez showed the least antimicrobial activity against all the three microorganisms. Inhibition of microbial growth is related to the direct contact of microorganisms with the sealers. In conclusion AH Plus had significantly higher antimicrobial activity against *E. faecalis, C. albicans*, and *S. aureus*.

## 1. Introduction

Microbes and microbial products are the main etiologic factors of pulpitis and apical periodontitis. The main aim of endodontic therapy is to eliminate microorganisms from the root canal. Instrumentation, irrigation, and intracanal medicaments significantly reduce the population of microorganisms. However it does not completely eliminate the microorganisms from the root canal; hence, a good root canal filling material with antibacterial property would be beneficial in further reducing the number of residual microorganisms [1].

One of the major causes for failure of root canal treatment is the presence of facultative and resistant microbial species of the oral cavity like* Enterococcus faecalis *(*E. faecalis*),* Candida albicans* (*C. albicans*), and* Staphylococcus aureus* (*S. aureus*) [2].

A variety of microbes ranging from anaerobes, aerobes and fungi can cause root canal infection. Among the aerobic bacteria* Enterococcus faecalis* is most commonly detected from endodontic infections ranging from 24 to 77% [3]. Other than* Enterococcus faecalis* (*E. faecalis*),* Candida albicans* (*C. albicans*) and* Staphylococcus aureus* (*S. aureus*) are other aerobic organisms associated with root canal infections [2].

*E. faecalis* is associated with persistent periradicular lesions after root canal treatment [4].

Similarly* C. albicans,* a part of normal microbiota, is associated with failed endodontic therapy and may be considered a dentinophilic microorganism [5].

Extraradicular microbial biofilm of* S. aureus *on tissue or biomaterial surface is related to refractory periapical disease [6]. Hence we choose these organisms as our study parameter.

Various studies have been performed to evaluate antimicrobial activity of different endodontic sealers [2, 7–11]. Agar diffusion test (ADT) is one of the most frequently used methods; however the information obtained from ADT does not completely reflect the* in vitro* antimicrobial activities [12].

ADT is a relatively insensitive and semiquantitative technique and does not distinguish between bactericidal and bacteriostatic effects of an agent. The results of ADT are also highly influenced by solubility and diffusibility of the test agent through the agar, and, therefore, this test is not suitable to assay water insoluble materials [13]. This could be overcome by direct contact test (DCT) which was used in this study and was introduced by Weiss et al. It is a turbidometric method which detects the bactericidal and bacteriostatic effect of endodontic sealers and root end filling materials. DCT measures effect of direct and close contact between the test microorganism and the tested material on microbial viability, regardless of the solubility and diffusibility of the antimicrobial components. This test is a quantitative and reproducible assay that allows testing of insoluble material and can be used in standardized settings [14].

There are very few studies where four endodontic sealers with different chemical compositions had been tested for their antimicrobial property against* E. faecalis, C. albicans*, and* S. aureus*. Furthermore none of the earlier studies have evaluated the effect of iRoot SP against* S. aureus. *Thus in the present study we evaluated the antimicrobial activity of four root canal sealers: an epoxy resin-based sealer, AH Plus (Dentsply International Inc., York, PA); zinc oxide-eugenol based sealer, Tubliseal EWT (Sybron Endo Corporation, Orange, CA); polymethacrylate resin-based sealer, EndoRez (Ultradent, South Jordan, UT); calcium hydroxide-calcium silicate complex sealer, iRoot SP (Innovative Bioceramix, Vancouver, Canada) against* E. faecalis*,* C. albicans*, and* S. aureus* by DCT. By using DCT not only was the direct antimicrobial activity of the tested endodontic sealers assessed, but also components of endodontic sealers capable of diffusing into the medium were assessed and statistically compared.

## 2. Materials and Methods

### 2.1. Microbial Strains

Standard strains of* E. faecalis*, American type culture collection (ATCC 29212),* C. albicans* (ATCC 90028), and* S. aureus* (ATCC 25923) were used in this study. Microbes were grown aerobically from frozen stock in brain heart infusion (BHI) broth at 37°C for 18–20 hrs. Inoculum was prepared to 0.5 McFarland standard. Sealers were prepared in strict compliance with the manufacturer's recommendations.

### 2.2. Endodontic Sealers

The four root canal sealers used in this study are as follows.

#### 2.2.1. AH Plus (Dentsply International Inc., York, PA)

It is an epoxy resin-based sealer, AH Plus Paste A composed of bisphenol-A epoxy resin, bisphenol-F epoxy resin, calcium tungstate, zirconium oxide, silica, and iron oxide pigments and AH Plus Paste B composed of dibenzyldiamine, aminoadamantane, tricyclodecane-diamine, calcium tungstate, zirconium oxide, silica, and silicone oil.

#### 2.2.2. Tubliseal EWT (Sybron Endo Corporation, Orange, CA)

It is a zinc oxide-eugenol based sealer composed of a base paste containing zinc oxide, oleo resin, bismuth trioxide, thymol iodide, oils and waxes, and a catalyst paste having eugenol, polymerized resins, and annidalin.

#### 2.2.3. EndoRez (Ultradent, South Jordan, UT)

It is a polymethacrylate resin-based sealer composed of triethylene glycol dimethacrylate and bismuth chloride oxide.

#### 2.2.4. iRoot SP (Innovative Bioceramix, Vancouver, Canada)

It is a calcium hydroxide-calcium silicate complex sealer consisting of zirconium oxide, calcium silicates, calcium phosphate, calcium hydroxide, filler, and thickening agents.

### 2.3. Direct Contact Test (DCT)

Direct contact test collects data by recording the optical density (OD), a measurement of turbidity that is based on the kinetics of microbial growth, with the help of a spectrophotometer, in 96-well microtiter plates (Tarson, Calcutta, India). The kinetics of the outgrowth in each well is monitored at 600 nm at 37°C and recorded every 30 mins using temperature controlled microplate spectrophotometer (Versa Max, Molecular devices corporation, USA). Automixing prior to each reading ensured a homogenous bacterial cell suspension.

Microtiter plate was held vertically such that the plate was maintained perpendicular to the plane. The side walls of 12 wells were coated with an even and thin freshly mixed sealer (Group A wells). Special care was taken to avoid the flow of material to the bottom of the well which could interfere with the light path through the microplate wells and result in false readings.

After 20 mins, 10 *μ*L of microbial suspension with OD of 0.5 McFarland standard (approximately 1.5 × 10^8^ CFU/mL) was placed on the test material while the plate remained vertical. Wells were inspected for evaporation of suspension which occurred within 1 hour at 37°C. This ensured direct contact between the microbes and test material.

Brain heart infusion broth (245 *μ*L) was added to each of the Group A wells and gently automixed for 2 mins. 15 *μ*L was then transferred from Group A wells to an adjacent set of 12 wells containing fresh medium (215 *μ*L) designated as Group B. This resulted in two sets of 12 wells for each tested material containing an equal volume of liquid medium so that microbial outgrowth could be monitored both in the presence and in the absence of the tested material. Following the outgrowth of the microorganisms in the presence of the tested material (Group A wells) is equivalent to measuring the direct contact effect. Following microbial growth in the absence of the tested materials (Group B wells) measures the effect of only those components which diffuse into the liquid medium.

Two sets of 12 uncoated wells in the same microtiter plate served as positive control; that is, identical microbial inoculum was placed on the side wall of uncoated wells and processed. The negative control consisted of a set of 12 wells coated with the tested material but containing an equal volume of uninoculated fresh medium. The plate was placed for incubation at 37°C in microplate spectrometer. The kinetics of outgrowth in each well was monitored at 600 nm and recorded every 30 mins, for 18 hrs.

The values of the negative control wells were considered the baseline and subtracted from the respective experimental sets. The entire experiment was carried out under aseptic conditions and performed in triplicate to ensure reproducibility.

### 2.4. Data Analysis

Descriptive statistical analysis has been carried out in the present study. Results on continuous measurements are presented as mean ± SD (min–max) and results on categorical measurements are presented in number (%). Significance is assessed at 5% level of significance. Analysis of covariance (ANCOVA) was used to find the significance. Intragroup analysis was done using Bonferroni correction for post hoc test at confidence level of 95%. The statistical software, namely, SAS 9.2, SPSS 15.0, Stata 10.1, MedCalc 9.0.1, Systat 12.0, and R environment ver.2.11.1, was used.

## 3. Results

### 3.1. Antimicrobial Activity of Tested Sealers against* E. faecalis*

Of the four sealers tested, significant antimicrobial activity was observed for AH Plus and iRoot SP against the positive control (*P* < 0.05). While AH Plus showed superior property of inhibition of* E. faecalis* throughout the time period of 18 hours of incubation, iRoot plus showed growth inhibition till 8 hours of incubation, following which it showed logarithmic increase. Tubliseal EWT and EndoRez showed inhibition of* E. faecalis* only within the first 5 hours of incubation showing no statistically significant difference with positive control in the Group A well.

**Figure 1:**
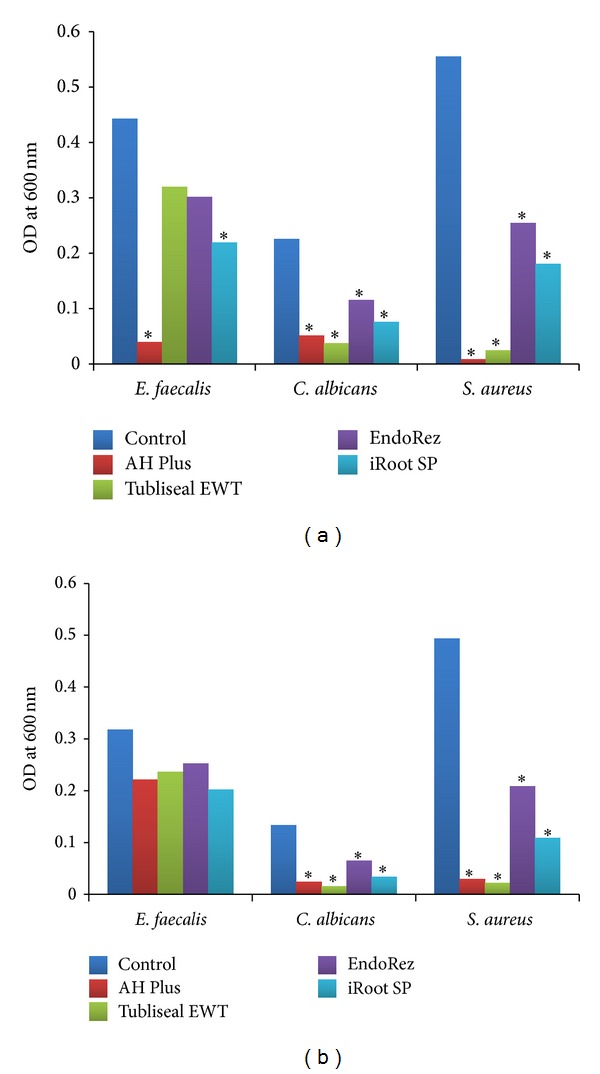
Comparison of time corrected mean microbial growth in (a) presence of tested sealers, A well and (b) absence of tested sealers, B well. The endodontic sealers showing significant antimicrobial property (*P* < 0.05) compared to the control are marked with ∗.

**Figure 2:**
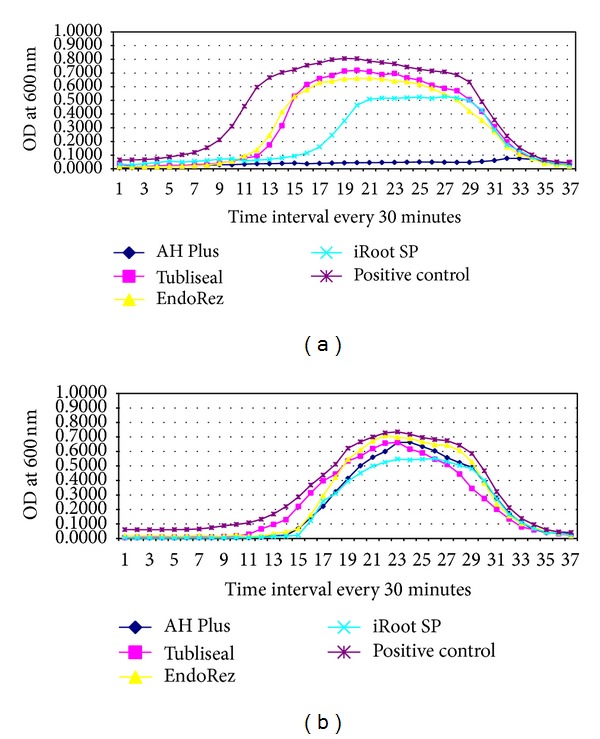
Line graph showing microbial growth curve of* E. faecalis *in (a) presence of tested sealers, A well and (b) absence of tested sealers, B well of the four different endodontic sealers. *x*-axis depicts time intervals at which the mean OD was recorded (at every 30 mins) and *y*-axis depicts mean corrected OD recorded at 600 nm. Each point on the growth curve is the average of the OD measured in 12 wells.

It was interesting to note that none of the sealers showed any significant diffusible antimicrobial activity in the Group B wells (Figures 1 and 2) against* E. faecalis*.

### 3.2. Antimicrobial Activity of Tested Sealers against* C. albicans*

All the four sealers had significant antimicrobial activity against* C. albicans* compared to the positive control (*P* < 0.05). Among the sealers, EndoRez showed a low level inhibition throughout the incubation time in the Group A well. EndoRez also showed poor direct and diffusible antimicrobial activity in the Group B wells compared to others, showing microbial growth after 6 hours of incubation.

**Figure 3:**
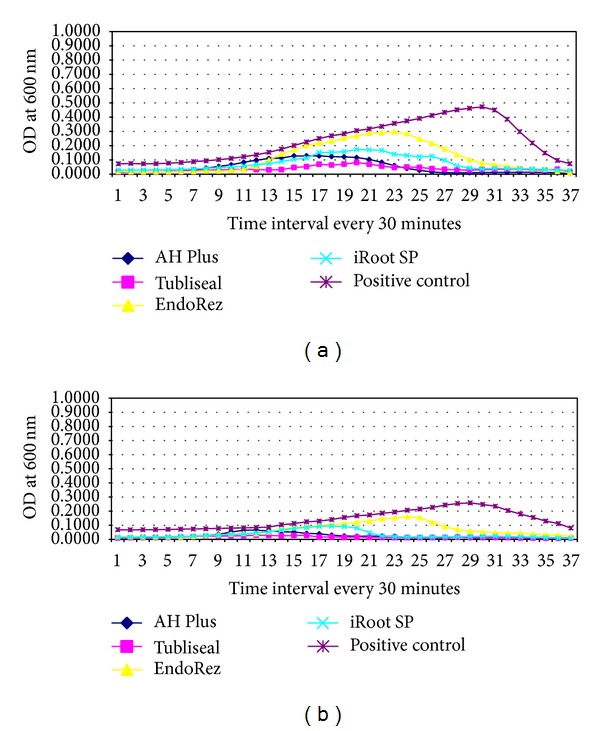
Line graph showing microbial growth curve of* C. albicans *in (a) presence of tested sealers, A well and (b) absence of tested sealers, B well of the four different endodontic sealers. *x*-axis depicts time intervals at which the mean OD was recorded (at every 30 mins) and *y*-axis depicts mean corrected OD recorded at 600 nm. Each point on the growth curve is the average of the OD measured in 12 wells.

However, AH Plus (*P* < 0.05), Tubliseal EWT (*P* < 0.05), and iRoot SP (*P* < 0.05) had significant diffusible antimicrobial activity against* C. albicans* (Figures 1 and 3) in Group B well.

### 3.3. Antimicrobial Activity of Tested Sealers against* S. aureus*

AH Plus (*P* < 0.05) and Tubliseal EWT (*P* < 0.05) had significant antimicrobial activity against* S. aureus* compared to others. Both inhibited* S. aureus* throughout the incubation period. iRoot SP and EndoRez showed significant rise in growth at 7 hours in Group A wells.

**Figure 4:**
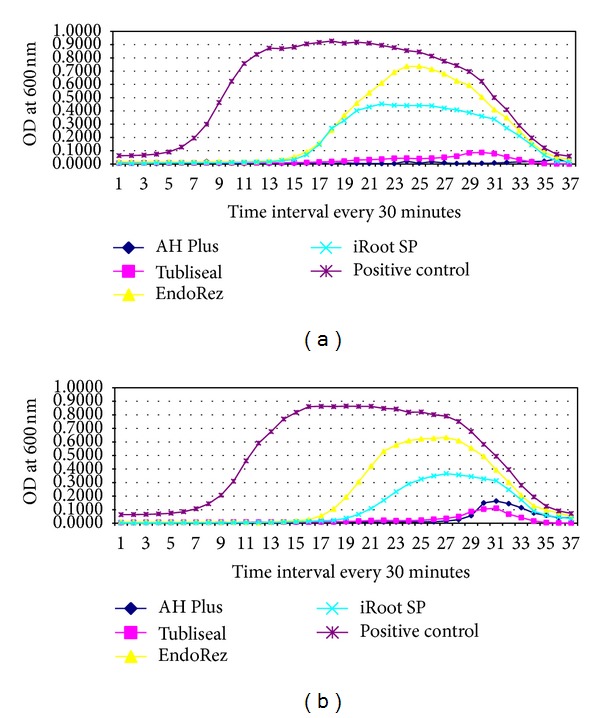
Line graph showing microbial growth curve of* S. aureus *in (a) presence of tested sealers, A well and (b) absence of tested sealers, B well of the four different endodontic sealers. *x*-axis depicts time intervals at which the mean OD was recorded (at every 30 mins) and *y*-axis depicts mean corrected OD recorded at 600 nm. Each point on the growth curve is the average of the OD measured in 12 wells.

AH Plus (*P* < 0.05) and Tubliseal EWT (*P* < 0.05) had significant diffusible antimicrobial activity against* S. aureus* (Figures 1 and 4) in Group B wells.

## 4. Discussion

The results of the present study showed AH Plus with the highest antimicrobial activity against* E. faecalis* followed by iRoot SP. AH Plus and Tubliseal EWT showed the highest antimicrobial activity against* C. albicans* and* S. aureus*. EndoRez showed the least antimicrobial activity against all the three test microorganisms as measured in direct contact. However, all the four sealers showed good diffusible property against* C. albicans *and* S. aureus *and poor diffusible property against* E. faecalis *(Figures 1, 2, 3, and 4).

These results are in accordance with other studies where fresh AH Plus and iRoot SP had antibacterial action against* E. faecalis* by time kill assay [15], ADT, DCT, and modified DCT [1, 2, 16, 17].

Literature search indicates that freshly mixed AH Plus had better antimicrobial activity than Tubliseal EWT against* E. faecalis* [1]. Our study corroborated these findings and AH Plus was a better antimicrobial compared to eugenol based Tubliseal EWT against* E. faecalis*. The antimicrobial effect of AH Plus can be attributed to small quantity of formaldehyde [18], epoxy, and amine ingredients released during the polymerization process [19]. Any other factor contributing to the superior performance of AH Plus remains a matter of speculation.

For the first time iRoot SP has been evaluated for its antimicrobial activity against* S. aureus*, where it shows potent antimicrobial activity. iRoot SP is a new calcium phosphate silicate based endodontic sealer and root end filling material [20]. The pH of iRoot SP, a bioceramic sealer [21], during setting is higher than 12. The alkaline pH of iRoot could be responsible for its antibacterial activity [22].

In our study iRoot SP showed significant antifungal activity against* C. albicans* which is in accordance with other studies [11]. The antimicrobial activity of iRoot SP sealer might be due to combination of high pH, hydrophilicity, and active calcium hydroxide diffusion.

Tubliseal EWT is a zinc oxide based sealer. Zinc oxide-eugenol based sealers have traditionally been the most commonly employed sealers during root canal treatment. They serve as the benchmark with which the other sealers are compared. In our study Tubliseal EWT has shown significant antimicrobial activity against* C. albicans *and* S. aureus, *which could be attributed to eugenol. Eugenol is a phenolic compound that acts on microorganisms by denaturation whereby the protein becomes nonfunctional. It is also effective against mycotic cells and vegetative forms [10]. The results of our study match previous studies done using different methodologies [1, 23, 24].

EndoRez is a methacrylate resin with hydrophilic properties [25]. EndoRez showed the least antimicrobial activity against all the three microorganisms in the present study. Studies on EndoRez have yielded contradictory results till date [1, 7, 16, 17, 26]. Hence further studies are required to clarify their status.

Literature search does not reveal too many studies where endodontic sealers with four different formulae were pitted against three different microorganisms considered to be resistant to endodontic treatment using advanced methodology like the direct contact test. Any sealer effective against these microorganisms will probably be effective against more susceptible microbes.

A caveat of our study is that we used DCT, which does not allow evaluation of microorganisms in biofilm conditions [22]. Microbes in direct contact with endodontic biomaterials (endodontic obturating materials and sealers) may form biofilm. Though endodontic biomaterial with antimicrobial property may reduce formation of biofilm and prove promising for successful root canal therapy, we could not address the effect of these biomaterials in a biofilm model. Further we did not look into CFU by culturing onto plate at different time intervals since we used an incubating plate reader, which takes reading at the specified time. Also due to technical constraints we could not look into the efficacies of these dental sealers against the anaerobic bacterias, which are majorly associated with endodontic infections.

To conclude, our study showed epoxy resin-based sealer AH Plus to have highest antimicrobial activity against both bacteria (*E. faecalis and S. aureus*) and yeast (*C. albicans*), while polymethacrylate resin-based sealer EndoRez showed the least antimicrobial activity against all the three microorganisms. However, these assessments are based on an* in vitro* aerobic culture technique which may not immediately reflect clinical efficacy* in vivo*. The results have to be corroborated on a biofilm model to better reflect clinical efficacy.
